# Evaluation of Changes in Grams of Sugar Sold After the Implementation of the Seattle Sweetened Beverage Tax

**DOI:** 10.1001/jamanetworkopen.2021.32271

**Published:** 2021-11-05

**Authors:** Lisa M. Powell, Julien Leider, Vanessa M. Oddo

**Affiliations:** 1 Division of Health Policy and Administration, University of Illinois Chicago School of Public Health, Chicago; 2 Institute for Health Research and Policy, University of Illinois Chicago, Chicago; 3 Department of Kinesiology and Nutrition, University of Illinois Chicago College of Applied Health Sciences, Chicago

## Abstract

**Importance:**

Adults and children routinely exceed recommended intake amounts of added sugars established by dietary guidelines. Taxes are used as a policy tool to reduce demand for sugar-sweetened beverages (SSBs) given consumption-related adverse health outcomes but may induce substitution to other sources of added sugars.

**Objective:**

To examine the extent to which changes in grams of sugar sold from taxed beverages may be offset by changes in grams of sugar sold from untaxed beverages, sweets, and stand-alone sugar after the implementation of the Seattle, Washington, Sweetened Beverage Tax (SBT) on January 1, 2018.

**Design, Setting, and Participants:**

This study used difference-in-differences analyses to examine changes in grams of sugar sold from taxed and untaxed products in Seattle compared with Portland, Oregon, at year 1 and year 2 post tax. This study used Nielsen scanner data from supermarkets and mass merchandise as well as grocery, drug, convenience, and dollar stores on unit sales and measurements for beverage and food product universal product codes (UPCs) for each site for the pretax period (January 8-December 30, 2017) and the corresponding weeks in year 1 post tax (2018) and in year 2 post tax (2019). Nutritional analyses assessed grams of sugar for each UPC. The analytical balanced sample included 1326 taxed beverage UPCs, 239 untaxed beverage UPCs, 2054 sweets UPCs, and 81 stand-alone sugar UPCs. Statistical analysis was performed from January to August 2021.

**Exposures:**

Implementation of the Seattle SBT.

**Main Outcomes and Measures:**

Changes in grams of sugar sold from taxed beverages, untaxed beverages, sweets, and stand-alone sugar.

**Results:**

At both year 1 and year 2 post tax in Seattle compared with Portland, grams of sugar sold from taxed beverages decreased 23% (year 2 posttax ratio of incidence rate ratios [RIRR] = 0.77; 95% CI, 0.73-0.80). Sugar sold from untaxed beverages increased at year 1 post tax by 4% (RIRR = 1.04; 95% CI, 1.00-1.07) with no change at year 2 post tax. Sugar sold from sweets increased by 4% at both year 1 and year 2 post tax (year 2 posttax RIRR = 1.04; 95% CI, 1.03-1.06). There were no changes in stand-alone sugar sold.

**Conclusions and Relevance:**

This study using difference-in-differences analysis found a net 19% reduction in grams of sugar sold from taxed SSBs at year 2 post tax after accounting for changes in sugar sold from untaxed beverages, sweets, and stand-alone sugar. These results suggest that SSB taxes may effectively yield permanent reductions in added sugars sold from SSBs in food stores.

## Key Points

**Question:**

To what extent were reductions in grams of sugar sold from taxed beverages offset by increases in sugar sold from potential substitution to untaxed beverages and foods after the 2018 implementation of the Sweetened Beverage Tax in Seattle, Washington?

**Findings:**

In this study of beverage and food universal product codes using difference-in-differences analysis, after accounting for potential substitution to untaxed beverages, sweets, and stand-alone sugar, there were net reductions in grams of sugar sold from taxed sugar-sweetened beverages of 18% in year 1 post tax and 19% in year 2 post tax.

**Meaning:**

Findings of this study suggest that sugar-sweetened beverage taxes may yield permanent reductions in added sugars sold from sugar-sweetened beverages in food stores.

## Teaser

This study uses difference-in-differences analysis to assess the association between introducing a sugar-sweetened beverage tax and net added sugars sold from sugar-sweetened beverages, after accounting for potential substitution to other key sources of added sugars.

## Introduction

Added sugars, including those from sugar-sweetened beverages (SSBs), contribute to adverse health outcomes such as obesity, type 2 diabetes, cardiovascular disease, and poor dental health.^1,2,3,4^ The US 2020-2025 Dietary Guidelines for Americans recommend that added sugars contribute less than 10% of daily caloric intake.^5^ More than 50% of adults and 65% of children do not meet these guidelines.^6,7^ Moreover, adults who exceed the recommendations of the US Dietary Guidelines for Americans consume approximately 4 times more added sugars than their counterparts who meet the recommendations.^6^ For children aged 2 to 19 years, 5.9% of calories are obtained from added sugars among those who meet the US Dietary Guidelines for Americans guidelines compared with 18.5% among those who do not meet the guidelines.^7^

SSBs are the leading source of added sugars intake in the US diet, contributing, on average, 37% for men, 28% for women, 21% for children aged 2 to 5 years, 29% for children aged 6 to 11 years, and 37% for children aged 12 to 19 years.^6,7^ In addition, added sugars from tea and coffee beverages are key contributors for adults (17%) and youths aged 12 to 19 years (8%), whereas added sugars from flavored milk are a notable source for younger children (5%).^6,7^

A key objective and leading health indicator for Healthy People 2030 is to “reduce consumption of added sugars by people aged 2 years and over.”^8^ Given the substantial contribution of SSBs to added sugars intake, curbing SSB consumption is an important public health goal. Notably, SSB taxes are increasingly being used worldwide as a policy tool aimed at reducing consumption. These taxes are typically applied to soda, fruit, energy and sports drinks, and sweetened teas/coffees; however, to date, such taxes have usually excluded sweetened/flavored milk with added sugars and do not apply to non–sugar-sweetened beverages, such as juice or tea/coffee with sugar added separately by consumers. Thus, it is important to understand the extent to which consumers may substitute from taxed to untaxed beverages with added sugars. It is also important to understand the extent to which any potential reductions in added sugars intake from SSBs may be offset by increases in added sugars intake from potential substitution to high-sugar foods. For example, combined sweets categories of sweet bakery products, candy, and other desserts are shown to contribute 20% to 27% of the total added sugars intake for adults and 27% to 37% for children.^6,7^

Recent evaluations have reported that SSB taxes are generally effective in reducing demand; for example, taxes that raise prices by 20% are found, on average, to reduce demand for taxed beverages by approximately 20% to 27%.^9,10,11^ Many evaluations also have assessed the cross-price SSB tax impacts on substitution to untaxed beverages and have reported mixed results.^9^ No studies to date have assessed the extent of substitution to calorically sweetened/flavored milk, although several have assessed substitution to milk and generally found no increases in demand.^12,13,14,15,16,17^ In addition, a limited number of evaluations have examined the association between SSB taxes and substitution to sugary foods, such as sweets. An evaluation of the Philadelphia, Pennsylvania, tax (levied on both SSBs and artificially sweetened beverages) found that, in year 1 post tax, there was no change in dollar sales or grams of sweets sold in Philadelphia compared with the reference site of Baltimore, Maryland.^18^ Another Philadelphia study that used receipts obtained from consumers exiting small independent food stores also found no substitution to sweets and found that overall grams of sugar purchased from SSBs and high-sugar foods decreased 34.1% in year 2 post tax.^19^ A recent evaluation of the Seattle, Washington, tax found that sales increased by 4% and calories sold from sweets increased by 3% in year 1 post tax compared with the reference site of Portland, Oregon.^20^ However, a study of the Mexico tax on SSBs and energy-dense foods using household purchase scanner data found that the reduced calories and sugar from purchases of taxed products in year 1 post tax were offset by increases in calories and sugar from purchases of untaxed food and beverage categories.^21^

This study aims to understand the potential outcomes associated with an SSB tax on reducing the net added sugars sold from SSBs after accounting for potential substitution to other key sources of added sugars. A difference-in-differences (DID) approach is used to examine longer-run year 2 posttax changes in grams of sugar sold from taxed SSBs, untaxed beverages, sweets, and stand-alone sugar itself in Seattle, Washington, compared with Portland, Oregon, after the implementation on January 1, 2018, of the 1.75-cent-per-ounce Seattle Sweetened Beverage Tax (SBT) on SSBs with at least 40 kcal/12 ounces. To our knowledge, this is the first study to evaluate the potential outcomes associated with a US SSB tax in a broad set of food stores on net grams of sugar sold from products that contribute to the majority of added sugars intake in the US diet.

## Methods

For this study using DID analysis, we obtained retail scanner data on unit sales and unit measurements (eg, fluid ounces or grams) of sugary beverages, sweets, and stand-alone sugar products in Seattle and Portland from Nielsen scanner data. As described previously,^14^ the data included all sales at stores in Nielsen’s sample of supermarkets and mass merchandise as well as grocery, drug, chain and nonchain convenience, and dollar stores, which was estimated to cover 45% of food store sales in Seattle; Portland was chosen as a comparison site based on Mahalanobis distance matching.^22^ This study follows the Strengthening the Reporting of Observational Studies in Epidemiology (STROBE) reporting guideline and was approved by the University of Illinois Chicago Institutional Review Board, which also waived informed patient consent because this study did not involve human participants.

The analyses in this study drew on data from 1 year pretax (January 8 to December 30, 2017) and the corresponding periods for year 1 and year 2 post tax. Statistical analysis was performed from January to August 2021. Of 11 264 nonalcoholic beverage universal product codes (UPCs) sold in Seattle and Portland across these 3 periods, sweetened status overall and for milks specifically could not be determined for 105 and 13 UPCs, respectively; of the remainder, 5468 were sugary beverages. After balancing on UPCs sold in both sites in all 3 periods, 1784 UPCs remained. The analytical sample for sugary beverages included 1565 UPCs, representing 78.47% of volume sold after excluding 170 store-brand UPCs (nutritional data could not be obtained as stores were deidentified) and 49 other UPCs for which sugar content could not be determined. Of 26 195 sweet UPCs sold in Seattle and Portland over the same periods, 6696 remained after balancing. Nutritional data were not available for 1181 store-brand UPCs, and nutritional data were researched for the top 80% of nonstore-brand sweet UPCs by units sold in Seattle and Portland, leaving an analytical sample of 2054 sweet UPCs representing 65.39% of units sold. In addition, of 196 stand-alone sugar UPCs sold in Seattle and Portland over these periods, the analytical sample included 81 after balancing, which represented 93.05% of units sold. Coverage for volume or units sold of all 3 analytical samples (ie, sugary beverages, sweets, and stand-alone sugar products) was similar across sites and times.

Beverages were classified as taxed or untaxed based on Seattle’s ordinance; classified by size as individual size (single item ≤1 L) and family size (>1 L or multipack); and classified by type as taxed juice drinks, soda, sports drinks, energy drinks, and tea/coffee, untaxed sweetened (flavored/unflavored) milk, and untaxed SSBs with less than 40 kcal/12 ounces. Sweets were classified by type as candy/confections, frozen desserts, cookies, and other sweets (eg, cakes, pies, muffins, pastries, brownies, and doughnuts). Sugar itself (eg, white/brown sugar) was classified as a single stand-alone sugar category.

Using a research protocol, beverage calorie content and type as well as sugar content for both beverages and sweets were obtained from Label Insight, the United States Department of Agriculture Food Composition databases, and internet research under the direction of a registered dietitian.^23,24^ Beverage size, sweet type, and information on the per-unit weight in grams of stand-alone sugar UPCs were obtained from the Nielsen data. Grams of sugar sold for each beverage UPC were computed as the number of ounces sold times grams of sugar per ounce, which included all sugar in the case of sugary beverages and only added sugars in the case of sweetened milks. Grams of sugar sold for each sweet and stand-alone sugar UPC were computed as the number of units sold times the sugar content per unit based on nutritional research for sweets or the product gram weight for stand-alone sugar. eAppendix 1 in the Supplement presents details on the nutritional coding process.

### Statistical Analysis

The DID estimates of changes in grams of sugar sold in year 1 and year 2 posttax implementation for sugary beverages, sweets, and stand-alone sugar in Seattle compared with Portland were computed from Poisson models of the following form:

E(*Sugar_ist_* | *Time*_2018_*_t_*, *Time*_2019_*_t_*, *Seattle_s_*) = *exp* (β_0_ + β_1_*Time*_2018_*_t_* + β_2_*Time*_2019_*_t_* + β_3_*Seattle_s_*

**Figure 1.**
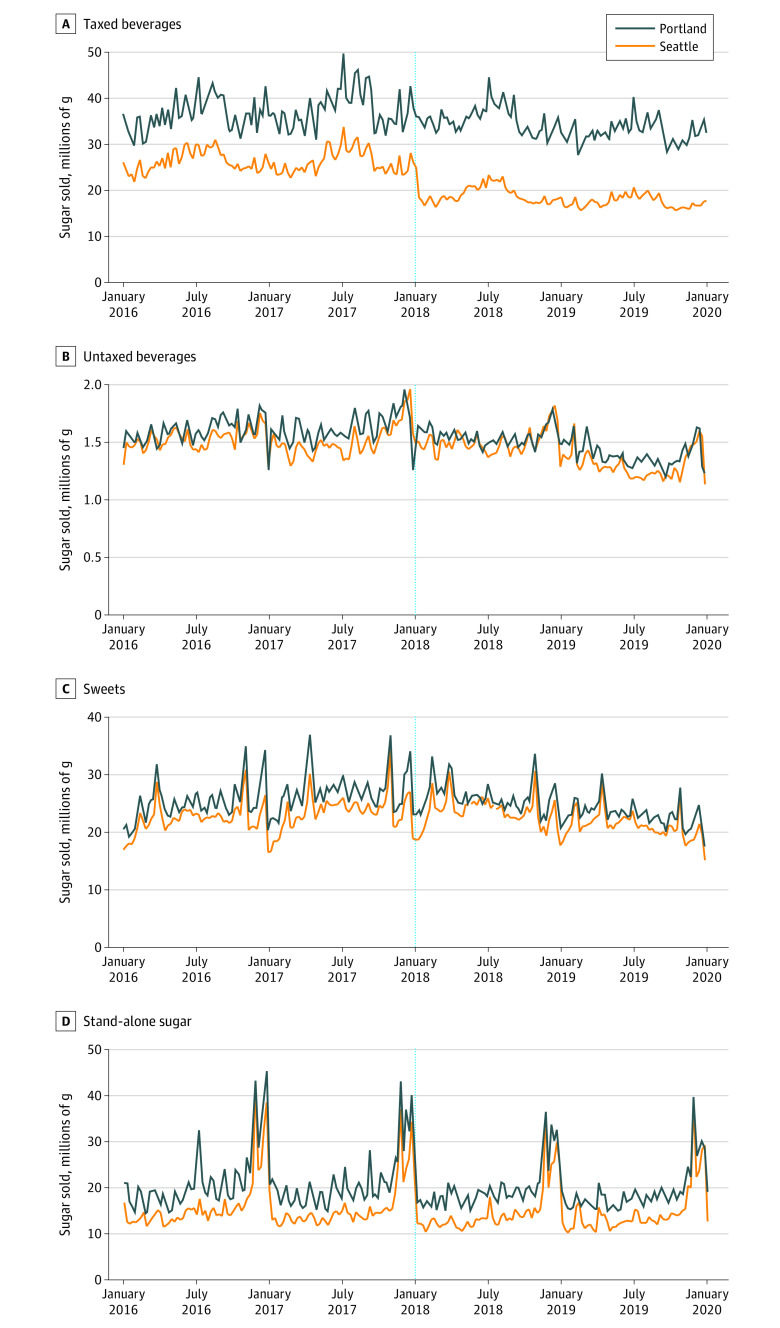
Sugar Sold in Seattle, Washington, and Portland, Oregon, From 2 Years Pretax Through 2 Years Post Tax

+ β_4_*Time*_2018_*_t_* × *Seattle_s_* + β_5_*Time*_2019_*_t_* × *Seattle_s_*)

where *Sugar_ist_* is grams of sugar sold of UPC *i* in site *s* and time *t*, *Time_2018t_* indicates observations from 2018 and *Time_2019t_* indicates observations from 2019, and *Seattle_s_* indicates observations from Seattle. Poisson pseudo maximum likelihood was used because parallel trends and tax effects were both expected to take a multiplicative form.^25^ The exponentiated interaction terms β_4_ and β_5_ are ratios of incidence rate ratios (RIRRs) showing the percentage change in grams of sugar sold in Seattle compared with Portland. Models were estimated with robust standard errors clustered on UPC. With only 1 intervention and 1 comparison site, we were unable to cluster at the site level and hence standard errors may be underestimated.^26,27^ Statistical significance was determined at the .05 level based on 2-sided tests, and only estimates that were significant are reported along with their corresponding 95% CIs. Analyses were conducted in Stata/SE, version 15.1 (StataCorp LLC). eAppendix 2 in the Supplement presents a more detailed description of our DID estimation methods, including test results supporting the parallel trends assumption, which is required for interpreting results from DID models. Additionally, Figure 1 provides evidence from pretax trends that supports the parallel trends assumption between Seattle and Portland for each of the 4 outcomes.

## Results

The analytical balanced sample included 1326 taxed beverage UPCs, 239 untaxed beverage UPCs, 2054 sweets UPCs, and 81 stand-alone sugar UPCs. The summary statistics presented in Table 1 report mean (SD) kilograms of sugar sold per UPC in Seattle and Portland pretax and in year 1 and year 2 posttax implementation. The mean (SD) kilograms of sugar sold for taxed SSB UPCs was 1006 (2919) in the pretax period in Seattle, which decreased to 669 (1840) at the year 2 posttax implementation. The mean (SD) kilograms of sugar sold pretax in Seattle was highest for soda at 1888 (4849) and lowest for tea/coffee drinks at 357 (708). The mean (SD) kilograms of sugar sold for untaxed beverages was 322 (626); among sweets, it was highest for frozen desserts at 746 (919) and lowest for other sweets at 405 (537). In addition, mean (SD) kilograms of sugar sold was the highest of all categories of UPCs for stand-alone sugar at 9838 (23 959). The pretax mean kilograms of sugar sold across product categories was consistently higher in Portland compared with Seattle.

**Table 1.** Kilograms of Sugar Sold in Seattle, Washington, and Portland, Oregon, Pretax to Year 2 Post tax^a^

Variable	UPCs, No.	Mean (SD)					
		Seattle			Portland		
		Pretax^b^	Post tax		Pretax^b^	Post tax	
			Year 1^c^	Year 2^d^		Year 1^c^	Year 2^d^
Taxed beverages	1326	1006 (2919)	729 (1954)	669 (1840)	1437 (4081)	1352 (3874)	1246 (3708)
Juice drinks	347	562 (973)	439 (781)	385 (721)	776 (1331)	704 (1240)	623 (1116)
Soda	413	1888 (4849)	1283 (3174)	1209 (2998)	2818 (6641)	2663 (6305)	2511 (6078)
Sports drinks	134	1060 (1214)	769 (891)	669 (797)	1354 (1505)	1224 (1351)	1065 (1204)
Energy drinks	133	828 (1752)	718 (1486)	617 (1366)	1191 (3338)	1195 (3236)	1040 (2949)
Tea/coffee	299	357 (708)	289 (535)	274 (508)	444 (943)	421 (871)	396 (831)
Untaxed beverages	239	322 (626)	321 (659)	278 (613)	345 (669)	331 (670)	297 (623)
Sweetened milk^e^	176	404 (707)	407 (745)	357 (695)	430 (754)	417 (758)	377 (705)
SSBs	63	93 (159)	79 (136)	60 (112)	107 (187)	93 (152)	74 (128)
Sweets	2054	591 (887)	590 (1081)	527 (910)	672 (1015)	646 (1249)	576 (986)
Candy/confections	994	579 (874)	594 (1233)	506 (884)	650 (960)	652 (1481)	542 (919)
Frozen desserts	490	746 (919)	719 (941)	650 (947)	773 (1033)	701 (931)	653 (994)
Cookies	278	558 (1102)	551 (1139)	538 (1185)	659 (1338)	620 (1266)	597 (1343)
Other sweets^f^	292	405 (537)	400 (523)	386 (538)	586 (771)	561 (721)	540 (767)
Stand-alone sugar	81	9838 (23 959)	9286 (23 957)	9236 (24 541)	13 155 (39 855)	12 405 (38 799)	11 869 (38 177)

Abbreviations: SSBs, sugar-sweetened beverages; UPCs, universal product codes.

^a^ Total kilograms of sugar sold per UPC were computed for each site and year. Means and SD shown were computed across UPCs. Sample sizes shown are for the number of UPCs.

^b^ The pretax period was defined as January 8, 2017, to December 30, 2017.

^c^ The year 1 posttax period was defined as January 7, 2018, to December 29, 2018.

^d^ The year 2 posttax period was defined as January 6, 2019, to December 28, 2019.

^e^ For sweetened milks, only added sugars, not naturally occurring sugar, are counted.

^f^ Other sweets included, for example, cakes, pies, muffins, pastries, brownies, and doughnuts.

**Figure 2.**
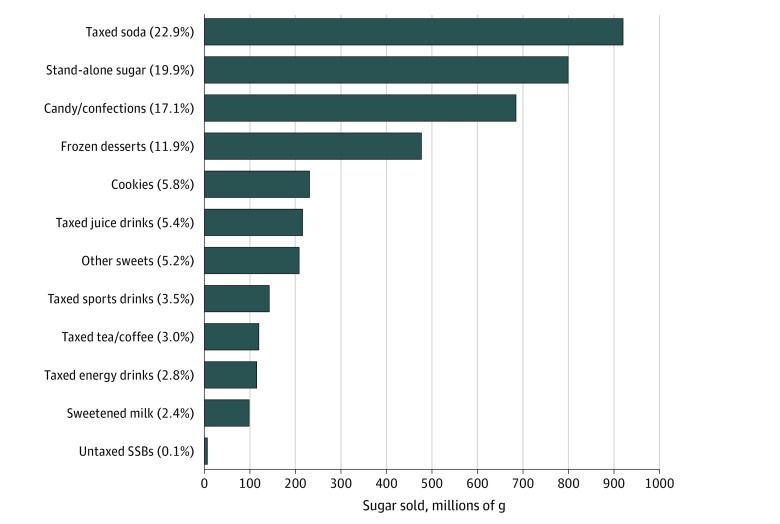
Distribution of Grams of Sugar Sold, Pretax Period, Seattle, Washington Total grams of sugar sold in Seattle from January 8, 2017, to December 30, 2017, are shown for each beverage and sweet type and for stand-alone sugar. Each value in parentheses preceding each bar is the percentage of grams of sugar represented of the total grams of sugar across all beverage and sweet types and stand-alone sugar. For sweetened milks, only added sugars, not naturally occurring sugar, are counted. Other sweets included, for example, cakes, pies, muffins, pastries, brownies, and doughnuts. SSBs indicates sugar-sweetened beverages.

Figure 2 shows the distribution of total grams of sugar sold in Seattle in the pretax period across the beverage, sweets, and stand-alone sugar categories included in this study. eAppendix 3 in the Supplement provides calculation details. Among these categories, taxed soda was the largest single contributor to sugar sold, accounting for almost one-quarter (22.9% [918 of 4010 million grams]). Next, stand-alone sugar accounted for 19.9% (797 of 4010 million grams), followed by substantial contributions from 2 categories of sweets: 17.1% (684 of 4010 million grams) from candy/confections and 11.9% (476 of 4010 million grams) from frozen desserts. The remaining categories of beverages and sweets each accounted for less than 6% of sugar sold.

### Changes in Grams of Sugar Sold From Taxed Beverages

Table 2 reports the DID results for changes in grams of sugar sold from taxed beverages, untaxed beverages, sweets, and stand-alone sugar (including subcategories) in Seattle compared with Portland from the pretax period to year 1 and year 2 posttax implementation. The results show that there was a 23% decrease in sugar sold from taxed beverages, which was sustained through year 2 posttax implementation (RIRR = 0.77; 95% CI, 0.74-0.80 at year 1 post tax; RIRR = 0.77; 95% CI, 0.73-0.80 at year 2 post tax). Across beverage categories, reductions in sugar sold were, on average, larger for family-sized (31%; RIRR = 0.69; 95% CI, 0.65-0.73) compared with individual-sized taxed beverages (10%; RIRR = 0.90; 95% CI, 0.87-0.93) at year 2 post tax. By taxed SSB category, the largest reduction of sugar sold was for soda, down 28% (RIRR = 0.72; 95% CI, 0.67-0.77), with a 35% (RIRR = 0.65; 95% CI, 0.60-0.70) reduction for family-sized soda compared with only a 5% (RIRR = 0.95; 95% CI, 0.91-0.99) reduction for individual-sized soda at year 2 post tax.

**Table 2.** Pretax to Posttax Changes in Grams of Sugar Sold in Seattle, Washington, Compared With Portland, Oregon^a^

Variable	UPCs, No.^b^			RIRR (95% CI)					
	Total	Individual size	Family size	Total		Individual size		Family size	
				Year 1 post tax	Year 2 post tax	Year 1 post tax	Year 2 post tax	Year 1 post tax	Year 2 post tax
Taxed beverages	1326	813	513	0.77 (0.74-0.80)	0.77 (0.73-0.80)	0.89 (0.87-0.92)	0.90 (0.87-0.93)	0.70 (0.66-0.73)	0.69 (0.65-0.73)
Juice drinks	347	205	142	0.86 (0.82-0.90)	0.85 (0.80-0.91)	1.03 (0.96-1.10)	1.02 (0.94-1.12)	0.81 (0.76-0.86)	0.80 (0.74-0.86)
Soda	413	182	231	0.72 (0.68-0.77)	0.72 (0.67-0.77)	0.94 (0.90-0.97)	0.95 (0.91-0.99)	0.65 (0.61-0.69)	0.65 (0.60-0.70)
Sports drinks	134	95	39	0.80 (0.77-0.83)	0.80 (0.77-0.83)	0.79 (0.76-0.83)	0.80 (0.76-0.84)	IS	IS
Energy drinks	133	114	19	0.86 (0.83-0.90)	0.85 (0.80-0.91)	0.87 (0.84-0.90)	0.86 (0.81-0.92)	IS	IS
Tea/coffee	299	217	82	0.85 (0.81-0.90)	0.86 (0.81-0.91)	0.89 (0.84-0.94)	0.88 (0.81-0.95)	0.82 (0.76-0.88)	0.84 (0.76-0.92)
Untaxed beverages	239	147	92	1.04 (1.00-1.07)	1.00 (0.95-1.07)	1.06 (1.02-1.09)	1.03 (0.97-1.10)	1.03 (0.98-1.08)	1.00 (0.92-1.08)
Sweetened milk^c^	176	98	78	1.04 (1.00-1.08)	1.01 (0.95-1.07)	1.06 (1.02-1.10)	1.04 (0.97-1.11)	1.03 (0.98-1.09)	1.00 (0.93-1.08)
SSBs	63	49	14	0.98 (0.90-1.08)	0.93 (0.81-1.07)	IS	IS	IS	IS
Sweets	2054	NA	NA	1.04 (1.02-1.05)	1.04 (1.03-1.06)	NA	NA	NA	NA
Candy/confections	994	NA	NA	1.02 (1.00-1.05)	1.05 (1.03-1.07)	NA	NA	NA	NA
Frozen desserts	490	NA	NA	1.06 (1.04-1.09)	1.03 (1.00-1.06)	NA	NA	NA	NA
Cookies	278	NA	NA	1.05 (1.02-1.08)	1.06 (1.03-1.10)	NA	NA	NA	NA
Other sweets^d^	292	NA	NA	1.03 (1.01-1.06)	1.03 (1.00-1.07)	NA	NA	NA	NA
Stand-alone sugar	81	NA	NA	1.00 (0.91-1.11)	1.04 (0.91-1.19)	NA	NA	NA	NA

Abbreviations: IS, insufficient sample size; NA, not applicable; RIRR, ratio of incidence rate ratios; SSBs, sugar-sweetened beverages; UPC, universal product code.

^a^ Estimates are shown from Poisson difference-in-differences regression models with robust SEs clustered on UPC. The pretax period was defined as January 8, 2017, to December 30, 2017; the year 1 posttax period was defined as January 7, 2018, to December 29, 2018; and the year 2 posttax period was defined as January 6, 2019, to December 28, 2019.

^b^ Sample sizes are shown in terms of the number of UPCs; the number of observations for analyses equaled the number of UPCs times 6 (2 sites times 3 time periods). Estimates are not shown when they would be based on fewer than 50 UPCs.

^c^ For sweetened milks, only added sugars, not naturally occurring sugar, are counted.

^d^ Other sweets included, for example, cakes, pies, muffins, pastries, brownies, and doughnuts.

### Changes in Grams of Sugar Sold From Untaxed Products

In terms of substitution to untaxed products, while there was a 4% (RIRR = 1.04; 95% CI, 1.00-1.07) increase in grams of sugar sold from untaxed beverages overall in Seattle compared with Portland in the first year post tax, this increase was not sustained at year 2 posttax implementation. The results also showed that sugar sold from sweets increased similarly by 4% at year 1 (RIRR = 1.04; 95% CI, 1.02-1.05) and year 2 (RIRR = 1.04; 95% CI, 1.03-1.06) post tax. In addition, there was not a significant change in grams of sugar sold from stand-alone sugar in Seattle compared with Portland at either year 1 or year 2 posttax implementation.

### Net Changes in Grams of Sugar Sold

The DID estimates of changes in grams of sugar sold from untaxed beverages, sweets, and stand-alone sugar along with the baseline total grams of sugar from these sources were used to assess the extent to which substitution to these products may have offset the 23% reduction in grams of sugar sold from taxed beverages. After accounting for the 4% increase in grams of sugar sold from untaxed beverages at year 1 post tax and the 4% increase from sweets at both year 1 and year 2 post tax, the reduction in sugar sold from taxed SSBs in Seattle compared with Portland was offset by 20% at year 1 post tax and by 19% at year 2 post tax, resulting in a net reduction in grams of sugar sold from SSBs of 18% at year 1 post tax and 19% at year 2 post tax. The calculation details are available in eAppendix 4 in the Supplement.

## Discussion

A key public health objective is to reduce added sugars intake. While a robust body of evidence supports that SSB taxes are associated with reductions in the demand for SSBs, limited and mixed evidence exists on their impact on changes in demand for other sugary products^18,19,20,21^; hence, the potential role of SSB taxes in reducing overall added sugars intake is less understood. To better understand the extent to which SSB taxes can effectively contribute to the goal of sugar intake reduction, this study drew on UPC-level scanner data from a broad range of store types to provide evidence on the net change in grams of sugar sold after the implementation of the Seattle SBT across the major food and beverage products contributing to added sugars intake in the US diet. The study results showed that grams of sugar sold decreased from taxed SSBs by 23% at year 1 and year 2 post tax, increased by 4% from both untaxed beverages and sweets at year 1 post tax and from sweets only by 4% at year 2 post tax, and no changes were observed from stand-alone sugar at either year 1 or year 2 post tax. Thus, after accounting for potential substitution to untaxed beverages, sweets, and stand-alone sugar, the key findings showed net reductions in grams of sugar sold from taxed SSBs of 18% in year 1 post tax and 19% in year 2 post tax.

The reductions in grams of sugar sold from taxed SSBs were shown to largely stem from substantial reductions from family-sized taxed beverages, particularly soda. This result is consistent with previous scanner-based demand study findings in Seattle^14^ and other jurisdictions,^12,15^ which have found relatively large reductions in volume sold for soda. This outcome may be expected given that soda is among the cheapest SSBs and hence experiences among the largest price increases from the taxes.^12,14,28^ In addition, of key relevance is the fact that sodas are substantial contributors to the volume sold of SSBs^14^ and are particularly high-sugar SSBs.^29^

This study found that grams of sugar sold from untaxed beverages increased by 4% at year 1 post tax but that this substitution did not persist at year 2 post tax. The change at year 1 post tax stemmed from substitution to sweetened milk, which has not been assessed in previous evaluations. It should be noted that because the focus of this study was to capture changes in grams of sugar from products with added sugars, we did not assess substitution to unsweetened juice. The World Health Organization has highlighted the health risks associated with free sugars and has recommended their intake be reduced^30^; thus, it has been suggested that unsweetened juice be taxed.^9^ However, given that most (including for Seattle)^12,14,15,16,17^ but not all^13,31^ tax evaluations have not found increases in demand for unsweetened juice (ie, substitution), it is unlikely that SSB taxes would lead to increases in free sugar intake from this source. In addition, given no evidence of cross-border shopping after the implementation of the Seattle SBT,^14^ we do not expect changes in sugar sold from purchases in the border area.

The study results showing that grams of sugar sold from sweets increased 4% and persisted for 2 years post tax suggest that the reduction in grams of sugar sold from SSBs was somewhat offset. Previous demand models that have assessed cross-price effects between SSBs and sweets have found mixed results on whether they are substitutes.^32,33,34,35,36^ In addition, results from recent tax evaluations that have assessed substitution to sweets are also mixed.^18,19,20,21^

### Limitations

To our knowledge, this is the first study to examine changes following the implementation of a US SSB tax in grams of sugar from key contributors to added sugars intake sold in a broad range of different types of food stores; nonetheless, it is subject to several limitations. First, because this study uses store scanner data, it is unable to address potential differences in changes in consumption patterns across populations. Second, due to confidentiality restrictions, we do not have information on store name, type, and location and thus were not able to assess differential changes based on these characteristics. Third, this study does not cover grams of sugar sold in nonstore venues (eg, restaurants, workplace cafeterias, and vending machines), and thus the results may not generalize to overall changes in net added sugars intake from all sources. Fourth, the results from Seattle may not generalize to other local tax jurisdictions.

## Conclusions

To date, there is limited and mixed evidence from SSB tax evaluations on the extent to which reductions in sugar sold associated with reductions in SSB demand may be offset by potential substitution to untaxed beverages and foods that are key contributors to added sugars intake. The findings of this study of a net 19% year 2 posttax reduction in grams of sugar sold from taxed SSBs, after accounting for changes in sugar sold from untaxed beverages, sweets, and stand-alone sugar, suggest that SSB taxes are associated with permanent reductions in added sugars sold from SSBs in food stores and thus may reduce associated health harms.

## Notes

Supplement. **eAppendix 1.** Coding Process for Nutrition Information of Beverages and Sweets **eAppendix 2.** Difference-in-Differences Models **eAppendix 3.** Calculation of Total Grams of Sugar Sold **eAppendix 4.** Calculation of Net Change in Grams of Sugar Sold
eReferences
