# Supplementary material for: Evaluation of Changes in Grams of Sugar Sold After the Implementation of the Seattle Sweetened Beverage Tax
Source: JAMA Netw Open. 2021 Nov 5;4(11):e2132271. doi: 10.1001/jamanetworkopen.2021.32271 (PMC8571660; doi:10.1001/jamanetworkopen.2021.32271)
Supplement: Supplement. — eAppendix 1. Coding Process for Nutrition Information of Beverages and Sweets eAppendix 2. Difference-in-Differences Models eAppendix 3. Calculation of Total Grams of Sugar Sold eAppendix 4. Calculation of Net Change in Grams of Sugar Sold eReferences [file jamanetwopen-e2132271-s001.pdf]

Supplemental Online Content

Powell LM, Leider J, Oddo VM. Evaluation of changes in grams of sugar sold after the implementation of the Seattle Sweetened Beverage Tax. *JAMA Netw Open*. 2021;4(11):e2132271. doi:10.1001/jamanetworkopen.2021.32271

eAppendix 1. Coding Process for Nutrition Information of Beverages and Sweets

eAppendix 2. Difference-in-Differences Models

eAppendix 3. Calculation of Total Grams of Sugar Sold

eAppendix 4. Calculation of Net Change in Grams of Sugar Sold

eReferences

This supplemental material has been provided by the authors to give readers additional information about their work.

© 2021 Powell LM et al. *JAMA Network Open*.

eAppendix 1. Coding Process for Nutrition Information of Beverages and Sweets

Beverages

A registered dietitian (RD) reviewed the search protocol with the student coder, after which the student coder completed a training set of 50 UPCs that was reviewed by the RD. Additionally, we established a product review protocol and implemented several quality assurance checks. The RD first reviewed 10% of the UPCs entered by the student coder. Then, the RD searched the databases for any products the coder could not find. Next, she reviewed the assigned beverage category, beverage sweetener type, and sweetened milk indicator for discrepancies and made changes, as necessary. Finally, after data were received from the RD, checks were programmed to verify that there were no inconsistencies between assigned beverage categories and whether the beverage was categorized as sugar-sweetened, artificially sweetened, or unsweetened.

Sweets

Similar to our protocol for beverages, the RD first reviewed the search protocol with the student coder and then the student coder completed a training set of 100 UPCs. The RD independently coded the same 100 products to ensure consistency.

Additionally, we established a product review protocol and implemented several quality assurance checks. The RD first reviewed 25% of the UPCs entered by the student coder. Then, the RD searched the databases for any products the coder could not find. Next, she checked the nutrition information for any products with more than 500 calories per serving, more than 15 grams of saturated fat per serving, more than 450 mg of sodium per serving, or more than 40g of sugar per serving. Finally, the RD reviewed the products where the total package size reported in Label Insight (or other database) was different from that reported in Nielsen. The RD ensured information was entered for the package size identified in the Nielsen data where possible; where information on that specific size was not available, information was filled in for another size and the UPC was flagged so that analytical computations could appropriately adjust for this. In total, the RD reviewed more than one-third of the UPCs.

We also programmed several checks, which we conducted after receiving the final file from the RD. When there were differences in the total package size based on nutritional coding versus the Nielsen data, we verified that they were appropriately flagged, and checked the magnitude of any differences. In instances where the total package size was recorded in ounces, we checked for differences between the total size computed from the serving size in grams and the total number of servings and this recorded size. Finally, we checked the distribution of the nutritional variables. For any case of a suspected error, we verified the data with the RD.

eAppendix 2. Difference-in-Differences Models

Difference-in-differences (DID) models comparing changes from the pretax period (January 8-December 30, 2017) to the year 1 (January 7-December 29, 2018) and year 2 (January 6-December 28, 2019) posttax periods in Seattle, Washington (intervention site), and Portland, Oregon (comparison site), were used to estimate the potential impact of the Seattle Sweetened Beverage Tax (SBT) on changes in sugar sold from sugary beverages, sweets, and stand-alone sugar. Portland was chosen as the comparison site out of the four largest municipalities in each of Washington and Oregon based on Mahalanobis distance matching on population size, median household income, the percentage of the population below 125% of the poverty line, the percentage of the population that was non-Hispanic black or Hispanic, and the percentage of the population that was non-Hispanic Asian.

Interpretation of results from DID models relies on the parallel trends assumption, that changes in sugar sold in Seattle and Portland would have been the same in the absence of the SBT. Because the parallel trends assumption relates to counterfactual outcomes, it cannot be directly tested, but parallel trends in the pretax period were tested to provide evidence on the potential validity of this assumption. Specifically, weekly sugar sold per capita[1] was computed in each site (Seattle and Portland), and interaction terms for site by month in linear regression models with robust standard errors were tested using Wald tests. Sugar sold was computed on a per capita basis for these tests because they relied on weekly data aggregated across UPCs; the analytical models, which could not be used to test parallel trends as they aggregated yearly time periods, did not aggregate across UPCs and thus did not rely on sugar

© 2021 Powell LM et al. *JAMA Network Open*.

sold computed on a per capita basis. These tests revealed no statistically significant differences in pretax trends for taxed (*P*=.91) and untaxed (*P*=.47) sugary beverages, sweets (*P*>.99), and stand-alone sugar (*P*=.95). Figure 1 also demonstrates graphically that pretax trends were similar in the two sites.

Sugar sold was computed for each UPC in the analytical sample in each site and year; thus, the number of observations for analyses was equal to six times the number of UPCs. As shown in the main text of the paper, DID analyses were conducted using Poisson models of the form

$$\mathrm{E}(\mathrm{Sugar}_{ist} | Time_{2018t}, Time_{2019t}, Seattle_s) = \exp\,(\beta_0 + \beta_1 Time_{2018t} + \beta_2 Time_{2019t} + \beta_3 Seattle_s + \beta_4 Time_{2018t} * Seattle_s + \beta_5 Time_{2019t} * Seattle_s)$$

where $Sugar_{ist}$ is grams of sugar sold of UPC *i* in site *s* and time *t*, $Time_{2018t}$ and $Time_{2019t}$ indicate observations from 2018 and 2019, respectively, and $Seattle_s$ indicates observations from Seattle. Poisson models were used for this purpose because both parallel trends and treatment effects were expected to take a multiplicative form, and Poisson models have been shown to be appropriate in this setting and to avoid bias which can occur in log-linear regressions.[2] These are pseudo maximum likelihood models as the data are not expected to follow a Poisson distribution, so robust standard errors clustered on UPC were computed. The key parameters of interest from these models are the exponentiated interaction terms β_4 and β_5, which are ratios of incidence rate ratios (RIRR) showing the percentage change in sugar sold in Seattle relative to Portland.

© 2021 Powell LM et al. *JAMA Network Open*.

eAppendix 3. Calculation of Total Grams of Sugar Sold

The coverage of the analytical sample was adjusted for in computing total grams of sugar sold pretax in Seattle for each beverage and sweet type. For each beverage type, total grams of sugar sold was computed as the mean for the analytical sample times the balanced sample size, i.e., the analytical sample size plus the number of UPCs that were store-brand or otherwise missing nutritional data. The balanced sample size was 241 for untaxed sweetened milk, 63 for untaxed SSBs, 384 for taxed juice drinks, 486 for taxed soda, 134 for taxed sports drinks, 138 for taxed energy drinks, and 338 for taxed tea/coffee. For each sweet type, total grams of sugar were computed as the mean across the analytical sample times the analytical sample size, divided by the percentage of pretax units sold in Seattle for the analytical sample out of the balanced sample (76.36% for sweets overall, 84.10% for candy/confections, 76.81% for frozen desserts, 66.91% for cookies, and 57.05% for other sweets). No adjustment was necessary for stand-alone sugar because no UPCs had to be excluded due to missing nutritional data, so the total grams of sugar were simply computed as the mean times the analytical sample size.

© 2021 Powell LM et al. *JAMA Network Open*.

eAppendix 4. Calculation of Net Change in Grams of Sugar Sold

Based on the mean of 1006 kilograms of sugar multiplied by the balanced sample size of 1480 UPCs, nearly 1489 million grams of sugar were sold from taxed SSBs pretax in Seattle. Thus, the 23% reduction in grams of sugar sold from taxed SSBs at both year 1 and year 2 posttax corresponds to a reduction of more than 342 million grams of sugar sold. Similarly, based on the mean of 322 kilograms of sugar multiplied by the balanced sample size of 304 UPCs, nearly 98 million grams of sugar were sold from untaxed beverages pretax in Seattle, so the 4% increase in grams of sugar sold from untaxed beverages at year 1 posttax corresponds to an increase of nearly 4 million grams of sugar. Finally, based on the mean of 591 kilograms of sugar multiplied by the analytical sample size of 2054 UPCs and divided by the 76% of balanced sample units sold represented by the analytical sample, more than 1590 million grams of sugar were sold from sweets pretax in Seattle. The 4% increase in grams of sugar sold from sweets at both year 1 and year 2 posttax thus corresponds to an increase of nearly 64 million grams of sugar sold. The final net reduction in sugar sold is nearly 275 million grams at year 1 posttax and 279 million grams at year 2 posttax, indicating that substitution offset the reduction in grams of sugar sold due to the tax by 20% at year 1 posttax and 19% at year 2 posttax.

© 2021 Powell LM et al. *JAMA Network Open*.

eReferences

1. U.S. Census Bureau. 2014–2018 American Community Survey 5-Year Estimates. 2019. https://www2.census.gov/programs-surveys/acs/summary_file/2018/data/5_year_by_state/. Accessed April 15, 2020.
2. Ciani E, Fisher P. Dif-in-dif estimators of multiplicative treatment effects. *J Econom Methods.* 2019;8(1). doi:10.1515/jem-2016-0011

© 2021 Powell LM et al. *JAMA Network Open*.
